# Survey to evaluate clinical benefits and acceptance in international tele-intensive care unit collaboration

**DOI:** 10.1186/s13104-026-07884-6

**Published:** 2026-05-22

**Authors:** Franziska Lezius, Karin Steinecke, Anne Herholz, Stephen Schüürhuis, Vadym Terentyuk, Ivan Lisnyy, Khkmat Anvarov, Fathima Paruk, Jens-Peter König, Joachim Grosse, Andreas Edel, Daniel Hechler, Claudia D. Spies, Björn Weiss

**Affiliations:** 1 Department of Anesthesiology and Intensive Care Medicine, Charité, Universitätsmedizin Berlin, Corporate Member of Freie Universität Berlin and Humboldt Universität zu Berlin, Augustenburger Platz 1, 13353 Berlin, Germany; 2 Department of Infectious Diseases, Klinik Bavaria, Kreischa, Germany; 3 Institute of Biometry and Clinical Epidemiology, Charité, Universitätsmedizin Berlin, Corporate Member of Freie Universität Berlin and Humboldt-Universität zu Berlin, Berlin, Germany; 4 Bogomolets National Medical University, Kyiv, Ukraine; 5 Department of Anesthesiology and Intensive Care Medicine, National Cancer Institute, Kyiv, Ukraine; 6 Republican Research Centre of Emergency Medicine and Central Asian University, Tashkent, Uzbekistan; 7 Department of Critical Care, Steve Biko Academic Hospital, University of Pretoria, Pretoria, South Africa; 8 Department of Anesthesiology and Intensive Care Medicine, Sana Klinikum Lichtenberg, Berlin, Germany; 9 Department of Anesthesiology and Intensive Care Medicine, Evangelisches Krankenhaus Wesel, Wesel, Germany; 10 Centre for Biological Threats and Special Pathogens (ZBS), Robert Koch Institute, Berlin, Germany

**Keywords:** Telemedicine, Intensive care unit, Benefits, Acceptance, Data protection, Ethical concerns

## Abstract

**Objectives:**

Tele-intensive care unit (TICU) networks have evolved quickly in recent years and have proved their value in providing care for the critically ill. Objective measures to assess performance and quantify success e.g. clinical and economic outcome parameters are desirable but not easily accessible in international collaboration with very diverse clinical settings and legal prerequisites. Therefore, we initiated a study to evaluate the performance in our tele-intensive care unit network.

**Methods:**

We conducted an online survey addressing clinical staff at ten spoke sites in four countries connected to one TICU hub to assess subjective clinical benefit, acceptance and potential for improvement.

**Results:**

The majority of the 30 participants felt well supported clinically and personally, with a high acceptance and trust as well as a positive working relationship in tele-medical collaboration. Ethical principles and data protection were discovered to be persistently of utmost significance in tele-intensive care unit networks.

**Conclusion:**

International telemedicine in critical care is perceived positively without major differences between diverse health care systems when crossing borders, showing potential for improvement to guide future clinical and research approaches.

*Clinical trial registiry:* The study was registered on August 11th, 2023 with the German Clinical Trials Register (DRKS-IDDRKS00032246; https://www.drks.de/search/de/trial/DRKS00032246/details).

**Supplementary Information:**

The online version contains supplementary material available at 10.1186/s13104-026-07884-6.

## Introduction

Tele-intensive care units (TICU) have proved their value in providing care for the critically ill. Clinical outcome parameters i.e. mortality, hospital- and/ or intensive care unit (ICU)-length of stay and quality of care may be ameliorated by tele-medical support [1–8], adherence to quality indicators and best clinical practice in intensive care has been shown to be improved [9, 10] and intensive care units’ (ICU) costs may be decreased [1, 3, 5]. TICU collaboration has demonstrated to facilitate capacity building in clinical decision making, triage and transfers of critically ill patients in intensive care units [11] and physicians reported an increase in patients’ safety [12]. The recent COVID-19 pandemic accelerated the evolution of TICU networks (inter-) nationally [13, 14] aiming to share knowledge and attempting to compensate for the lack of trained intensive care specialists [15, 16]. Meanwhile despite rapid tele-medical development, data security and protection of ethical principles remain of foremost relevance in TICU collaboration [17–19]. Whereas clinical and economic benefits of TICU networks have been well shown in previous studies, the vast majority of these TICU collaborations operated in a single center [4, 7, 12], regionally [5, 8–11] or nationally [6, 14] therefore within the same or a comparable health care system. Contrarily, TICU networks crossing country borders are rare [3, 13, 20] and participants from hub and spoke sites are often closely culturally related and speak the same mother tongue [3, 20]. Evidence from multilateral TICU networks is scarce and objective measures to assess performance and quantify success (e.g. clinical and economic outcome parameters) are not easily accessible in international collaboration with very diverse clinical settings and legal prerequisites. Our tele-medical collaboration transitioned from a research driven regional critical care telemedicine program to an internationally operating TICU network, therefore demanded a thorough evaluation to assess success. In this survey-based study we investigate (i) the subjective clinical benefits of our TICU collaboration for patients and clinical staff at spoke sites, (ii) the trust and working relationship between the staff from hub and spoke sites as well as the interaction between the hub’s telemedical specialist and the patients at spoke sites, (iii) concerns regarding the ethical and data-protection framework at spokes sites, and (iv) the potential for improvement. Furthermore, we analyze (v) the differences regarding subjective clinical benefit, acceptance, and potential for improvement that might arise between institutions from different countries, with various languages, cultural backgrounds, legal prerequisites and clinical and economical resources, within our international TICU cooperation.

## Methods

### Study design

To assess subjective clinical benefit, acceptance, and potential for improvement, we used the sub-categories: (i) subjective benefit for patients and clinical staff, (ii) trust and working relationship, (iii) interaction with the patient, (iv) attitude of the users towards telemedical rounds and (v) potential for improvement of the TICU-Feedback-Tool. The TICU-Feedback-Tool was developed in a consensus process and explicitly tested for psychometric properties before starting routine use (supplement 1) [21]. We conducted an online survey addressing clinical personnel collaborating within our international TICU network. All, but one item is rated on a 5-point Likert scale. Potential for improvement was explored by using an open-ended question. The TICU-Feedback-Tool addressed all clinical staff namely nurses and physicians regularly participating in TICU consultations. The survey was carried out as an online questionnaire in English language from October 17, 2023 to December 6, 2023 using the statistical survey web app LimeSurvey. Participants were invited via emails: invitations were sent to one coordinator per spoke site who further distributed this email within the spoke site’s team, therefore the total number of recipients remains unknown. Participation was anonymous, no information on respondents’ demographics was collected to prevent identification of participants, encourage open and truthful answers and avoid social desirability bias. All participants were instructed on the purpose of the survey by a written information, and consent was obtained by actively agreeing on study and data protection information before entering the questionnaire. The study was approved by the local Institutional Review Board (No. EA2/163/23).

### TICU network set up

The TICU network comprises partner hospitals in four countries (Germany, South Africa, Ukraine, Uzbekistan) connected to one remote TICU hub at a tertiary university hospital in Germany. The TICU hub is staffed 24/7 by a board certified, experienced intensive care specialist. Tele-medical rounds are performed using a mobile device at the patients’ bedside providing high quality audio-visual sessions [22]. There is no continuous real time monitoring in place. The patients’ electronic medical record is documented on a password protected browser-based virtual care platform (Solo-Platform, Teladoc Health™). The entire technology is compliant with the Health Insurance Portability and Accountability Act (HIPAA) and the European Union’s General Data Protection Regulation (GDPR). Tele-consultations are pre-scheduled regularly (bi-weekly to daily from Monday to Friday, for a distinct day and time per spoke site at long term) depending on the facility’s needs. For emergencies, on demand-consultations are realized at any time 24/7 by the hub’s intensivist on call, who is contacted via phone or messenger by the spoke site’s physician on call to meet online if emergencies demand an urgent consultation. During regular working hours (Monday to Friday 7am to 5pm), case conferences with consultants from the whole range of medical specialties can be organized to address clinical questions surpassing the field of critical care. Consultations are carried out between hub and spoke sites, there were no consultations between different spoke sites. All TICU consultations are carried out in German or English and accompanied by a translator at the patients’ bedside if necessary.

### Statistical analysis

Data was analyzed with SPSS Statistics 27 and R (Version 4.1.2). Statistical analysis was conducted predominantly using methods of descriptive statistics. Categorical variables were analyzed using absolute and relative frequencies with 95% confidence intervals calculated using the Wilson method.

Given the limited sample size, nonparametric methods for factorial designs were utilized to compare seventeen TICU Feedback-Tool items across the countries. For each country and each item, relative effect estimates ($\:\widehat{{p}_{i}}$) with 95% confidence intervals and corresponding two-sided p-values were calculated in R (Version 4.1.2), employing the rankFD package (Version 0.1.1). Larger relative effect estimates suggest a tendency towards stronger subjective approval for aspects related to clinical benefits of tele-medical support. Specifically, $\:\widehat{{p}_{i}}$ > $\:\widehat{{p}_{j}}$ indicates that country $\:i$ tends to stronger approval as compared to country $\:j$.

All survey responses included in the analysis were complete, with no missing values. P-values below a significance level of ≤ 0,05 were considered significant. However, given the exploratory nature of this study, no additional p-value adjustments for multiplicity were applied beyond those inherent to the nonparametric multiple contrast tests for factorial designs. Hence, p-values were given as an orientation and are not interpreted as confirmatory.

## Results

Thirty clinical professionals from ten hospitals in all four countries (Germany: two hospitals/ eight participants, South Africa: two hospitals/ seven participants, Ukraine: five hospitals/ ten participants, Uzbekistan: one hospital/ five participants) completed the feedback survey with one to six participants per hospital. The survey was distributed via a professional network and further forwarded within institutions, therefore the total number of individuals who received the survey is unknown, hence a response rate cannot be provided. Essential hospital characteristics i.e. location, size and type of facility as well as ICU, the frequency of TICU rounds, staffing and the number of survey respondents are shown in Table 1.

**Table 1:** Hospital characteristics and survey respondents

No.*	Country	Income level	Loca-tion	Type of facility	Beds**	ICU/ faci-lity***	Type of ICU****	TICU round/ week*****	Physi-cian ratio******	Nurse ratio*******	Res-pon-dents********
1	Ukraine	Upper middle income	Urban	Regional/ provincial referral hospital	547	2	General/ cardiological/ surgical/ neuro/ trauma	1	≥ 1 per 10 beds	≥ 1 per 4 beds	1
2	Germany	High income	Peri- urban	Regional/ provincial referral hospital	355	1	General	5	< 1 per 10 beds	≥ 1 per 4 beds	3
3	Ukraine	Upper middle income	Urban	Regional/ provincial referral hospital	425	3	Pediatric	1	≥ 1 per 10 beds	< 1 per 4 beds	2
4	Ukraine	Upper middle income	Urban	National referral hospital	600	2	Surgical/ neuro/ oncological	1	≥ 1 per 10 beds	≥ 1 per 4 beds	5
5	Ukraine	Upper middle income	Urban	National referral hospital	520	5	Pediatric	1	< 1 per 10 beds	< 1 per 4 beds	1
6	Ukraine	Upper middle income	Urban	Regional/ provincial referral hospital	1000	> 5	General/ cardiological/ surgical/ neuro/ trauma/ oncological	1	≥ 1 per 10 beds	≥ 1 per 4 beds	1
7	Uzbe-kistan	Lower middle income	Urban	National referral hospital	650	> 5	General/ cardiological/ surgical/ neuro/ trauma/ other	3	≥ 1 per 10 beds	≥ 1 per 4 beds	5
8	Germany	High income	Urban	Specialty hospital	661	1	General/ cardiological/ surgical/ neuro/ trauma/ oncological/ other	2	≥ 1 per 10 beds	≥ 1 per 4 beds	5
9	South Africa	Upper middle income	Urban	Regional/ provincial referral hospital	600	> 5	Surgical/ trauma/ other	2	< 1 per 10 beds	≥ 1 per 4 beds	6
10	South Africa	Upper middle income	Urban	Regional/ provincial referral hospital	1899	> 5	Surgical/ other	1	< 1 per 10 beds	≥ 1 per 4 beds	1

*No. of Hospital, **Total no. of hospital beds, ***No. of ICUs per facility, ****Type(s) of participating ICU, *****No. of scheduled TICU rounds/ week, ******Ratio of physicians on duty per bed per shift (relative to the shift with the poorest ratio), *******Ratio of nurses on duty per bed per shift (relative to the shift with the poorest ratio), ******** No. of survey respondents

The results of the subjective benefit assessment for patients and clinical staff are displayed in Table 2. Twenty-four (80% [62.7%, 90.5%]) of the participants agreed or strongly agreed that patients’ safety was improved, twenty-five (83.7% [66.4%, 92.7%]) agreed or strongly agreed that consultations positively contributed to error avoidance and prevention whereas twenty-nine (96.7% [83.3%, 99.4%]) agreed or strongly agreed that the patients’ quality of care was improved. The majority, namely twenty-seven (90% [74.4%, 96.5%]), agreed or strongly agreed to feel well supported in taking difficult decisions and all clinical professionals answering the questionnaire agreed or strongly agreed to be able to refresh or acquire important medical knowledge by joining TICU rounds.

All clinical staff agreed or strongly agreed that there was always enough time to discuss their concerns during TICU rounds and twenty-six (86.7% [70.3%, 94.7%]) stated by agreeing or strongly agreeing that tele-medical visits were arranged at short notice if necessary.

**Table 2:** Subjective benefit assessment for patients and medical staff

Question	Strongly disagree *n* (%)	Disagree *n* (%)	Neutral *n* (%)	Agree *n* (%)	Strongly agree *n* (%)	Overall
Patient safety was improved/increased by the tele-medical visits	1 (3.3%)		5 (16.7%)	15 (50%)	9 (30%)	30 (100%)
The patients’ quality of care was improved by the tele-medical visits			1 (3.3%)	16 (53.3%)	13 (43.3%)	30 (100%)
The tele-medical visits positively contributed to error avoidance/error prevention in the treatment of my patients	1 (3.3%)		4 (13.3%)	17 (56.7%)	8 (26.7%)	30 (100%)
I felt well supported in taking difficult decisions by tele-medical visits			3 (10%)	16 (53.3%)	11 (36.7%)	30 (100%)
There was always enough time to discuss my concerns during tele-medical visits				14 (46.7%)	16 (53.3%)	30 (100%)
If necessary, tele-medical visits were arranged at short notice		3 (10%)	1 (3.3%)	14 (46.7%)	12 (40%)	30 (100%)
By taking part in tele-medical visit, I was able to refresh or acquire important medical knowledge				17 (56.7%)	13 (43.3%)	30 (100%)

Exploring the trust and working relationship twenty-eight (93.3% [78.7%, 98.2%]) of the clinical personnel agreed or strongly agreed that they were always able to address any uncertainties or treatment errors openly in tele-medical rounds, twenty-six (86.7% [70.3%, 94.7%]) agreed or strongly agreed that they implemented the treatment plans as discussed and all participants agreed or strongly agreed that collaboration with the tele-medical specialist was always friendly and constructive.

The interaction with the patient was evaluated positively with twenty-eight (93.3% [78.7%, 98.2%]) participants agreeing or strongly agreeing to the question “During the rounds, the tele-medical specialist treated my patients respectfully” and twenty-seven (90% [74.4%, 96.5%]) rating the item “My patients accepted the tele-medical specialist very well” with agree or strongly agree.

The items “I have ethical concerns about tele-medical visits” and “I have data protection concerns” about tele-medical visits” were rated diversely as displayed in Table 3. 16.7% [7.3%, 33.6%] (*n* = 5) agreed or strongly agreed that they had ethical concerns whereas almost one quarter (23.3% [11.8%, 40.9%], *n* = 7) of participants indicated by strongly agreeing or agreeing that they had data protection concerns. Contrarily, 70% [52.1%, 83.3%] (*n* = 21) strongly disagreed or disagreed to have ethical concerns and 60% [42.3%, 75.4%] (*n* = 18) strongly disagreed or disagreed to have data protection concerns.

**Table 3:** Ethical and data protection concerns

Question	Strongly disagree *n* (%)	Disagree *n* (%)	Neutral *n* (%)	Agree *n* (%)	Strongly agree *n* (%)	Overall
I have ethical concerns about tele-medical visits	7 (23.3%)	14 (46.7%)	4 (13.3%)	3 (10%)	2 (6.7%)	30 (100%)
I have data protection concerns about tele-medical visits	6 (20%)	12 (40%)	5 (16.7%)	5 (16.7%)	2 (6.7%)	30 (100%)

Overall > 90% of the clinical staff agreed or strongly agreed that tele-medical rounds helped them in treating their patients (*n* = 29, 96.7% [83.3%, 99.4%]), that they were satisfied with tele-medical rounds (*n* = 29, 96.7% [83.3%, 99.4%]) and that tele-medical consultations worked well (*n* = 29, 96.7% [83.3%, 99.4%]).

The majority namely twenty-two (73.3% [55.6%, 85.8%]) participants agreed or strongly agreed to the question on whether there was still potential for quality improvement of the tele-medical visits.

In the final open-ended question, one participant expressed the wish to have more access to dedicated specialists in various medical areas. One clinical staff desired to include ICU nurses from both partner facilities to discuss specific topics e.g. nutrition, infection control and patient’ hygiene inter-professionally, one participant opted for more case-based topic discussions within the network and two participants suggested to run tele-medical rounds bilaterally to spark interest and facilitate knowledge exchange.

Seventeen items of the TICU-Feedback Tool were compared between countries, all results are displayed in detail in Table 1S and Table 2S (supplement 2). For four questions in total, a significant difference was detected: namely participants from South Africa or Germany consented less strong or stronger relative to the mean distribution from all other countries.

With regard to patients’ and clinical staffs’ benefits participants from South Africa were less convinced relative to the mean distribution from all other countries that:

- The patients’ quality of care was improved by the tele-medical visits ($\:\widehat{\boldsymbol{p}}$ = 0.25 [0.18, 0.34]),
- The tele-medical visits positively contributed to error avoidance/ error prevention in the treatment of my patients ($\:\widehat{\boldsymbol{p}}$ = 0.23 [0.15, 0.35]),
- I felt well supported in taking difficult decisions by tele-medical visits ($\:\widehat{\boldsymbol{p}}$ = 0.30 [0.22, 0.39]).

German clinical personnel, evaluating the trust and working relationship, was more convinced relative to the mean distribution from all other countries that: The collaboration with the tele-medical specialist was always friendly and constructive ($\:\widehat{\boldsymbol{p}}$ = 0.65 [0.56, 0.72]). Table 4 presents the aforementioned differences between countries in detail (all items are displayed in supplement 2).

**Table 4:** Differences between countries

Item	Country	*n*	$\:\widehat{p}$	SE	95%-CI	Effect	*p*-value
The patients’ quality of care was improved by the telemedical visits	Germany	8	0.59	0.07	[0.44, 0.72]	0.12	0.60
	South Africa	7	0.25	0.04	[0.18, 0.34]	− 0.33	0.01*
	Ukraine	10	0.48	0.07	[0.35, 0.61]	− 0.02	0.99
	Uzbekistan	5	0.68	0.08	[0.51, 0.81]	0.23	0.21
The telemedical visits positively contributed to error avoidance/ error prevention in the treatment of my patients	Germany	8	0.54	0.06	[0.42, 0.66]	0.06	0.88
	South Africa	7	0.23	0.05	[0.15, 0.35]	− 0.35	0.04*
	Ukraine	10	0.53	0.07	[0.40, 0.66]	0.04	0.96
	Uzbekistan	5	0.69	0.08	[0.52, 0.82]	0.25	0.22
I felt well supported in taking difficult decisions by telemedical visits	Germany	8	0.63	0.07	[0.48, 0.75]	0.17	0.34
	South Africa	7	0.30	0.05	[0.22, 0.39]	− 0.27	0.02*
	Ukraine	10	0.37	0.07	[0.25, 0.51]	−0.17	0.31
	Uzbekistan	5	0.71	0.07	[0.55, 0.83]	0.27	0.12
The collaboration with the telemedical specialist was always friendly and constructive	Germany	8	0.65	0.04	[0.56, 0.72]	0.20	0.04*
	South Africa	7	0.36	0.08	[0.22, 0.53]	− 0.19	0.38
	Ukraine	10	0.45	0.07	[0.32, 0.59]	− 0.07	0.85
	Uzbekistan	5	0.55	0.08	[0.39, 0.70]	0.06	0.93

*p < 0.05, p-values equal to/ below a significance level of 0.05 were considered significant

## Discussion

Quickly growing international TICU networks crossing borders in recent years may grant valuable clinical support but also demand a thorough assessment of their success. Our survey reveals that clinical staff at spoke sites (i) perceives a positive subjective clinical benefit for themselves as well as their patients (ii) highly values the trust and working relationship with the hub-team (iii) rates the interaction between the TICU specialist and their patients positively, (iv) shows a favorable attitude towards telemedical rounds in general and (v) further contributes to the TICU collaboration by pointing out ethical and data protection concerns and by identifying highly appreciated potential for improvement.

In detail, the majority of participants perceived an improvement in quality of care, patient’ safety and error avoidance, therefore meeting the most crucial demands of TICU collaboration in an international approach beyond borders. These key findings are in line with prior research [12, 23].

Whereas Coletti et al. [12] found that 37% of residents rated nightly TICU support as a valuable educational experience, Kovacevic and colleagues [3] reported an improvement in various intensive care structures and processes. The TICU-team [3] achieved progress by establishing a weekly dedicated tele-education program between the staff of two tertiary care facilities in the USA as educators and one tertiary care hospital in Bosnia and Herzegovina as recipients. Both clinical teams shared the same native language. Our survey revealed that respondents felt well supported in decision-making and were able to acquire important medical knowledge by tele-consultations, similarly highly relevant when treating complex critically ill patients in intensive care. Hence, our results replicate prior studies’ findings. TICU-collaboration may facilitate knowledge exchange despite language barriers and different health care systems.

Moreover, our TICU-support was assessed as clinically appropriate and helpful, and delivered in a timely manner. Hence, rapid response with on demand consultations at short notice and sufficient time for discussions in this time critical medical area were ensured.

A respectful attitude of the TICU intensivist towards the patients and correspondingly a high acceptance from the patients’ perspective facilitates overcoming linguistic and cultural differences as one of the most relevant obstacles in telemedicine [24]. In a regional Dutch TICU program patients and relatives rated the tele-doctors’ attitudes as (very) respectful, felt their privacy protected and assessed the cooperation between hub and local intensivist as good [25]. Aligned with these findings, our collaboration was evaluated as trust worthy with a positive working relationship, thus realizing a friendly and constructive dialogue to address uncertainties openly.

The majority of participating clinicians at bedside implemented treatment plans as discussed in our TICU rounds and agreed that TICU consultations helped them in treating their patients. This is an important fact. A review on tele-intensive care revealed that TICU systems with decision-authority yielded better results [26]. Therefore, the fact that in our network treatment plans were implemented as discussed may potentially increase the chance of positive outcome effects.

Data protection and ethical considerations are important framework elements of telemedicine [19] and all participants’ countries of residence provide a legal framework for e-health/ telemedicine [27–30]. We found a considerable variation in these domains. Hence, despite rapid progress in telemedicine, data and privacy protection as well as ethical standards are non-negotiable fundamentals requiring careful consideration. Data security and ethical concerns may put a program at risk if not cautiously considered. According to WHO’s global survey on eHealth in 2010 only 25% of responding member states reported that their country had a national telemedicine policy or strategy with considerable differences between developed and developing countries [24]. Moreover, linguistic and cultural differences between patients and service providers as well as traditional approaches or indigenous practices and a lack of information and communication technology (ICT) literacy are found to be major barriers to the use of telemedicine [24]. Contrarily, no differences between countries were detected in our survey. Therefore, disparity may be primarily based on individual principles. Cultural background or national legal prerequisites may play a minor role. Qualitative research may yield more insight into ethical and data protection concerns in the future.

We found slight differences between participants from different countries regarding some items of the subjective clinical benefits that are not yet completely understood. They may not be explained due to mere country differences though, but rather due to the diversity of intensive care experience between the medical teams from different spoke sites. Junior physicians conducting TICU consultations may perceive a higher subjective benefit and knowledge gain compared to well experienced critical care specialists at remote sites.

Furthermore, the fact that all staff at hub and spoke sites are more at ease conducting TICU rounds in their native language may have contributed to the fact that German medical staff showed a tendency to rate the collaboration more often as friendly and constructive.

Finally, almost three quarters of the respondents identified potential for improvement. A bilateral inter-professional approach in TICU rounds sparks special interest and holds promise for future progress.

Limitations are inherent to surveys. At first, surveys do not allow to assess the direct benefit for patients as the main population of interest in telemedicine. In addition, the restricted number of participants - partially due to the nature of the survey distribution, which prevented us from determining exact response rates that were potentially low [31, 32], limited the ability to differentiate between facilities, and differences between countries should be interpreted as tendencies. Moreover, we were unable to prevent multiple participation and there was no information obtained on the respondents’ demographics including e.g. medical profession and level of professional experience as well as level of tele-medical experience to grant anonymity. Answers may be primarily personal opinion and may not be representative for an entire facility. Furthermore, spoke sites vary in structural prerequisites and medical standards resulting in different needs and demands. Besides, language barriers may render participation in online questionnaires more difficult.

Despite these limitations, our survey also has strengths. First, we used a validated instrument for TICU evaluation [21] that has been developed by TICU providers as well as recipients of tele-medical services. Secondly, the entire TICU network is subject to public funding which limits the risk of bias due to financial interests. The risk for social-desirability bias was mitigated by keeping the survey strictly anonymous. Finally, access to heterogeneous health care systems ranging from lower middle income to high income countries [33] including facilities in conflict-affected situations [34] increases the diversity of responses.

In summary, this survey showed that international telemedicine in critical care is perceived positively without differences when crossing country borders. Respondents indicate high acceptance and trust. A thorough communication of the underlying ethical framework and data security seems paramount for the success of a TICU collaboration.

## Supplementary Information

Supplementary Material 1.

Supplementary Material 2.

## Data Availability

Data is provided within the manuscript or supplementary information files.
