# Supplementary material for: Survey to evaluate clinical benefits and acceptance in international tele-intensive care unit collaboration
Source: BMC Res Notes. 2026 May 22;19:237. doi: 10.1186/s13104-026-07884-6 (PMC13198030; doi:10.1186/s13104-026-07884-6)
Supplement: Supplementary file 2 — Supplementary Material 2. [file 13104_2026_7884_MOESM2_ESM.docx]

Supplement 1: Table 1S and 2S

Table 1S: Frequency of TICU items by country

| **Question** | **Answer category** | **Germany**  **(n = 8)** | **South Africa**  **(n = 7)** | **Ukraine**  **(n = 10)** | **Uzbekistan**  **(n = 5)** | **Overall**  **(n = 30)** |
| --- | --- | --- | --- | --- | --- | --- |
| Patient safety was improved/increased by the telemedical visits | Strongly disagree | 0 (0%) | 1 (14.3%) | 0 (0%) | 0 (0%) | 1 (3.3%) |
|  | Disagree | 0 (0%) | 0 (0%) | 0 (0%) | 0 (0%) | 0 (0%) |
|  | Neutral | 0 (0%) | 2 (28.6%) | 3 (30.0%) | 0 (0%) | 5 (16.7%) |
|  | Agree | 4 (50.0%) | 4 (57.1%) | 5 (50.0%) | 2 (40.0%) | 15 (50.0%) |
|  | Strongly Agree | 4 (50.0%) | 0 (0%) | 2 (20.0%) | 3 (60.0%) | 9 (30.0%) |
| The patients' quality of care was improved by the telemedical visits | Strongly disagree | 0 (0%) | 0 (0%) | 0 (0%) | 0 (0%) | 0 (0%) |
|  | Disagree | 0 (0%) | 0 (0%) | 0 (0%) | 0 (0%) | 0 (0%) |
|  | Neutral | 0 (0%) | 1 (14.3%) | 0 (0%) | 0 (0%) | 1 (3.3%) |
|  | Agree | 3 (37.5%) | 6 (85.7%) | 6 (40.0%) | 1 (20.0%) | 16 (53.3%) |
|  | Strongly Agree | 5 (62.5%) | 0 (0%) | 4 (60.0%) | 4 (80.0%) | 13 (43.4%) |
| The telemedical visits positively contributed to error avoidance/error prevention in the treatment of my patients | Strongly disagree | 0 (0%) | 1 (14.3%) | 0 (0%) | 0 (0%) | 1 (3.3%) |
|  | Disagree | 0 (0%) | 0 (0%) | 0 (0%) | 0 (0%) | 0 (0%) |
|  | Neutral | 0 (0%) | 3 (42.9%) | 1 (10.0%) | 0 (0%) | 4 (13.3%) |
|  | Agree | 6 (75.0%) | 3 (42.9%) | 6 (60.0%) | 2 (40.0%) | 17 (56.7%) |
|  | Strongly Agree | 2 (25.0%) | 0 (0%) | 3 (30.0%) | 3 (60.0%) | 8 (26.7%) |
| I felt well supported in taking difficult decisions by telemedical visits | Strongly disagree | 0 (0%) | 0 (0%) | 0 (0%) | 0 (0%) | 0 (0%) |
|  | Disagree | 0 (0%) | 0 (0%) | 0 (0%) | 0 (0%) | 0 (0%) |
|  | Neutral | 0 (0%) | 1 (14.3%) | 1 (10.0%) | 0 (0%) | 3 (10.0%) |
|  | Agree | 3 (37.5%) | 6 (85.7%) | 6 (60.0%) | 1 (20.0%) | 16 (53.3%) |
|  | Strongly Agree | 5 (62.5%) | 0 (0%) | 3 (30.0%) | 4 (80.0%) | 11 (36.7%) |
| There was always enough time to discuss my concerns during telemedical visits | Strongly disagree | 0 (0%) | 0 (0%) | 0 (0%) | 0 (0%) | 0 (0%) |
|  | Disagree | 0 (0%) | 0 (0%) | 0 (0%) | 0 (0%) | 0 (0%) |
|  | Neutral | 0 (0%) | 0 (0%) | 0 (0%) | 0 (0%) | 0 (0%) |
|  | Agree | 2 (25.0%) | 4 (57.1%) | 6 (60.0%) | 2 (40.0%) | 14 (46.7%) |
|  | Strongly Agree | 6 (75.0%) | 3 (42.9%) | 4 (40.0%) | 3 (60.0%) | 16 (53.3%) |
| If necessary, telemedical visits were arranged at short notice | Strongly disagree | 0 (0%) | 0 (0%) | 0 (0%) | 0 (0%) | 0 (0%) |
|  | Disagree | 0 (0%) | 1 (14.3%) | 1 (10.0%) | 1 (20.0%) | 3 (10.0%) |
|  | Neutral | 0 (0%) | 1 (14.3%) | 0 (0%) | 0 (0%) | 1 (3.3%) |
|  | Agree | 2 (25.0%) | 4 (57.1%) | 6 (40.0%) | 2 (40.0%) | 14 (46.7%) |
|  | Strongly Agree | 6 (75.0%) | 1 (14.3%) | 3 (30.0%) | 2 (40.0%) | 12 (40.0%) |
| By taking part in telemedical visit, I was able to refresh or acquire important medical knowledge | Strongly disagree | 0 (0%) | 0 (0%) | 0 (0%) | 0 (0%) | 0 (0%) |
|  | Disagree | 0 (0%) | 0 (0%) | 0 (0%) | 0 (0%) | 0 (0%) |
|  | Neutral | 0 (0%) | 0 (0%) | 0 (0%) | 0 (0%) | 0 (0%) |
|  | Agree | 4 (50.0%) | 4 (57.1%) | 8 (80.0%) | 1 (20.0%) | 17 (56.7%) |
|  | Strongly Agree | 4 (50.0%) | 3 (42.9%) | 2 (20.0%) | 4 (80.0%) | 13 (43.3%) |
|  |  |  |  |  |  |  |

Continued

| **Question** | **Answer category** | **Germany**  **(n = 8)** | **South Africa**  **(n = 7)** | **Ukraine**  **(n = 10)** | **Uzbekistan**  **(n = 5)** | **Overall**  **(n = 30)** |
| --- | --- | --- | --- | --- | --- | --- |
| I was always able to address any uncertainties or treatment errors openly | Strongly disagree | 0 (0%) | 0 (0%) | 0 (0%) | 0 (0%) | 0 (0%) |
|  | Disagree | 0 (0%) | 0 (0%) | 0 (0%) | 0 (0%) | 0 (0%) |
|  | Neutral | 0 (0%) | 0 (0%) | 1 (10.0%) | 1 (20.0%) | 2 (6.7%) |
|  | Agree | 4 (50.0%) | 5 (71.4%) | 7 (70.0%) | 2 (40.0%) | 18 (60.0%) |
|  | Strongly Agree | 4 (50.0%) | 2 (28.6%) | 2 (20.0%) | 2 (40.0%) | 10 (33.3%) |
| I implemented the treatment plans as discussed in telemedical visits | Strongly disagree | 0 (0%) | 0 (0%) | 1 (10.0%) | 0 (0%) | 1 (3.3%) |
|  | Disagree | 0 (0%) | 0 (0%) | 0 (0%) | 0 (0%) | 0 (0%) |
|  | Neutral | 1 (12.5%) | 1 (14.3%) | 1 (10.0%) | 0 (0%) | 3 (10.0%) |
|  | Agree | 3 (37.5%) | 6 (85.7%) | 6 (60.0%) | 5 (100.0%) | 20 (66.7%) |
|  | Strongly Agree | 4 (50.0%) | 0 (0%) | 2 (20.0%) | 0 (0%) | 6 (20.0%) |
| The collaboration with the telemedical specialist was always friendly and constructive | Strongly disagree | 0 (0%) | 0 (0%) | 0 (0%) | 0 (0%) | 0 (0%) |
|  | Disagree | 0 (0%) | 0 (0%) | 0 (0%) | 0 (0%) | 0 (0%) |
|  | Neutral | 0 (0%) | 0 (0%) | 0 (0%) | 0 (0%) | 0 (0%) |
|  | Agree | 0 (0%) | 4 (57.1%) | 4 (40.0%) | 1 (20.0%) | 9 (30.0%) |
|  | Strongly Agree | 8 (100%) | 3 (42.9%) | 6 (60.0%) | 4 (80.0%) | 21 (70.0%) |
| During the rounds, the telemedical specialist treated my patients respectfully | Strongly disagree | 0 (0%) | 0 (0%) | 0 (0%) | 0 (0%) | 0 (0%) |
|  | Disagree | 0 (0%) | 0 (0%) | 1 (10.0%) | 0 (0%) | 1 (3.3%) |
|  | Neutral | 1 (12.5%) | 0 (0%) | 0 (0%) | 0 (0%) | 1 (3.3%) |
|  | Agree | 1 (12.5%) | 3 (42.9%) | 3 (30.0%) | 1 (20.0%) | 8 (26.7%) |
|  | Strongly Agree | 6 (75.0%) | 4 (57.1%) | 6 (60.0%) | 4 (80.0%) | 20 (66.7%) |
| My patients accepted the telemedical specialist very well | Strongly disagree | 0 (0%) | 0 (0%) | 0 (0%) | 0 (0%) | 0 (0%) |
|  | Disagree | 0 (0%) | 0 (0%) | 0 (0%) | 0 (0%) | 0 (0%) |
|  | Neutral | 1 (12.5%) | 1 (14.3%) | 1 (10.0%) | 0 (0%) | 3 (10.0%) |
|  | Agree | 3 (37.5%) | 5 (71.4%) | 5 (50.0%) | 1 (20.0%) | 14 (46.7%) |
|  | Strongly Agree | 4 (50.0%) | 1 (14.3%) | 4 (40.0%) | 4 (80.0%) | 13 (43.3%) |
| The telemedical rounds helped me in treating my patients | Strongly disagree | 0 (0%) | 0 (0%) | 0 (0%) | 0 (0%) | 0 (0%) |
|  | Disagree | 0 (0%) | 0 (0%) | 0 (0%) | 0 (0%) | 0 (0%) |
|  | Neutral | 0 (0%) | 1 (14.3%) | 0 (0%) | 0 (0%) | 1 (3.3%) |
|  | Agree | 3 (37.5%) | 5 (71.4%) | 6 (60.0%) | 1 (20.0%) | 15 (50.0%) |
|  | Strongly Agree | 5 (62.5%) | 1 (14.3%) | 4 (40.0%) | 4 (80.0%) | 14 (46.7%) |
| I have ethical concerns about telemedical visits | Strongly disagree | 4 (50.0%) | 1 (14.3%) | 1 (10.0%) | 1 (20.0%) | 7 (23.3%) |
|  | Disagree | 3 (37.5%) | 5 (71.4%) | 5 (50.0%) | 1 (20.0%) | 14 (46.7%) |
|  | Neutral | 1 (12.5%) | 0 (0%) | 2 (20.0%) | 1 (20.0%) | 4 (13.3%) |
|  | Agree | 0 (0%) | 1 (14.3%) | 1 (10.0%) | 1 (20.0%) | 3 (10.0%) |
|  | Strongly Agree | 0 (0%) | 0 (0%) | 1 (10.0%) | 1 (20.0%) | 2 (6.7%) |
|  |  |  |  |  |  |  |

Continued

| **Question** | **Answer category** | **Germany**  **(n = 8)** | **South Africa**  **(n = 7)** | **Ukraine**  **(n = 10)** | **Uzbekistan**  **(n = 5)** | **Overall**  **(n = 30)** |
| --- | --- | --- | --- | --- | --- | --- |
| I have data protection concerns about telemedical visits | Strongly disagree | 4 (50.0%) | 0 (0%) | 1 (10.0%) | 1 (20.0%) | 6 (20.0%) |
|  | Disagree | 3 (37.5%) | 3 (42.9%) | 5 (50.0%) | 1 (20.0%) | 12 (40.0%) |
|  | Neutral | 1 (12.5%) | 1 (14.3%) | 2 (20.0%) | 1 (20.0%) | 5 (16.7%) |
|  | Agree | 0 (0%) | 3 (42.9%) | 1 (10.0%) | 1 (20.0%) | 5 (16.7%) |
|  | Strongly Agree | 0 (0%) | 0 (0%) | 1 (10.0%) | 1 (20.0%) | 2 (6.7%) |
| Overall, I am satisfied with telemedical rounds | Strongly disagree | 0 (0%) | 0 (0%) | 0 (0%) | 0 (0%) | 0 (0%) |
|  | Disagree | 0 (0%) | 0 (0%) | 0 (0%) | 0 (0%) | 0 (0%) |
|  | Neutral | 0 (0%) | 0 (0%) | 1 (10.0%) | 0 (0%) | 1 (3.3%) |
|  | Agree | 4 (50.0%) | 5 (71.4%) | 4 (40.0%) | 2 (40.0%) | 15 (50.0%) |
|  | Strongly Agree | 4 (50.0%) | 2 (28.6%) | 5 (50.0%) | 3 (60.0%) | 14 (46.7%) |
| All in all, I think there is still potential for quality improvement of the telemedical visits | Strongly disagree | 0 (0%) | 0 (0%) | 0 (0%) | 0 (0%) | 0 (0%) |
|  | Disagree | 2 (25.0%) | 0 (0%) | 0 (0%) | 0 (0%) | 2 (6.7%) |
|  | Neutral | 2 (25.0%) | 2 (28.6%) | 1 (10.0%) | 1 (20.0%) | 6 (20.0%) |
|  | Agree | 2 (25.0%) | 5 (71.4%) | 7 (70.0%) | 1 (20.0%) | 15 (50.0%) |
|  | Strongly Agree | 2 (25.0%) | 0 (0%) | 2 (20.0%) | 3 (60.0%) | 7 (23.3%) |
|  |  |  |  |  |  |  |

Table 2S: Results from the nonparametric factorial designs obtained by rankFD

| **Item** | | **Country** | **n** | $\hat{\boldsymbol{p}}$ | **SE** | **95%-CI** | **Effect** | **p-value** |
| --- | --- | --- | --- | --- | --- | --- | --- | --- |
| Patient safety was improved/ increased by the telemedical visits | | Germany | 8 | 0.63 | 0.07 | [0.50, 0.75] | 0.18 | 0.28 |
|  |  | South Africa | 7 | 0.28 | 0.06 | [0.18, 0.40] | -0.30 | 0.059 |
|  |  | Ukraine | 10 | 0.41 | 0.07 | [0.28, 0.56] | -0.11 | 0.63 |
|  |  | Uzbekistan | 5 | 0.67 | 0.08 | [0.51, 0.80] | 0.23 | 0.24 |
| The patients' quality of care was improved by the telemedical visits | | Germany | 8 | 0.59 | 0.07 | [0.44, 0.72] | 0.12 | 0.60 |
|  |  | South Africa | 7 | 0.25 | 0.04 | [0.18, 0.34] | -0.33 | **0.01*** |
|  |  | Ukraine | 10 | 0.48 | 0.07 | [0.35, 0.61] | -0.02 | 0.99 |
|  |  | Uzbekistan | 5 | 0.68 | 0.08 | [0.51, 0.81] | 0.23 | 0.21 |
| The telemedical visits positively contributed to error avoidance/ error prevention in the treatment of my patients | | Germany | 8 | 0.54 | 0.06 | [0.42, 0.66] | 0.06 | 0.88 |
|  |  | South Africa | 7 | 0.23 | 0.05 | [0.15, 0.35] | -0.35 | **0.04*** |
|  |  | Ukraine | 10 | 0.53 | 0.07 | [0.40, 0.66] | 0.04 | 0.96 |
|  |  | Uzbekistan | 5 | 0.69 | 0.08 | [0.52, 0.82] | 0.25 | 0.22 |
| I felt well supported in taking difficult decisions by telemedical visits | | Germany | 8 | 0.62 | 0.07 | [0.48, 0.75] | 0.17 | 0.34 |
|  |  | South Africa | 7 | 0.30 | 0.05 | [0.22, 0.39] | -0.27 | **0.02*** |
|  |  | Ukraine | 10 | 0.37 | 0.07 | [0.25, 0.51] | -0.17 | 0.31 |
|  |  | Uzbekistan | 5 | 0.71 | 0.07 | [0.55, 0.83] | 0.27 | 0.12 |
| There was always enough time to discuss my concerns during telemedical visits | | Germany | 8 | 0.60 | 0.08 | [0.45, 0.74] | 0.14 | 0.54 |
|  |  | South Africa | 7 | 0.44 | 0.09 | [0.28, 0.61] | -0.08 | 0.89 |
|  |  | Ukraine | 10 | 0.43 | 0.08 | [0.29, 0.58] | -0.10 | 0.75 |
|  |  | Uzbekistan | 5 | 0.53 | 0.10 | [0.34, 0.71] | 0.04 | 0.99 |
| If necessary, telemedical visits were arranged at short notice | | Germany | 8 | 0.69 | 0.07 | [0.55, 0.81] | 0.26 | 0.13 |
|  |  | South Africa | 7 | 0.35 | 0.08 | [0.22, 0.52] | -0.19 | 0.35 |
|  |  | Ukraine | 10 | 0.47 | 0.08 | [0.33, 0.62] | -0.04 | 0.97 |
|  |  | Uzbekistan | 5 | 0.48 | 0.11 | [0.27, 0.69] | -0.03 | 0.998 |
| By taking part in telemedical visit, I was able to refresh or acquire important medical knowledge | | Germany | 8 | 0.51 | 0.08 | [0.35, 0.66] | 0.01 | 0.999 |
|  |  | South Africa | 7 | 0.47 | 0.08 | [0.32, 0.64] | -0.04 | 0.99 |
|  |  | Ukraine | 10 | 0.36 | 0.07 | [0.24, 0.50] | -0.19 | 0.22 |
|  |  | Uzbekistan | 5 | 0.66 | 0.08 | [0.48, 0.80] | 0.21 | 0.31 |
| I was always able to address any uncertainties or treatment errors openly | | Germany | 8 | 0.60 | 0.08 | [0.43, 0.74] | 0.13 | 0.63 |
|  |  | South Africa | 7 | 0.50 | 0.08 | [0.34, 0.65] | -0.005 | 1.000 |
|  |  | Ukraine | 10 | 0.42 | 0.07 | [0.29, 0.57] | -0.10 | 0.71 |
|  |  | Uzbekistan | 5 | 0.48 | 0.12 | [0.26, 0.71] | -0.02 | 0.999 |
| I implemented the treatment plans as discussed in telemedical visits | | Germany | 8 | 0.64 | 0.09 | [0.45, 0.79] | 0.19 | 0.39 |
|  |  | South Africa | 7 | 0.41 | 0.06 | [0.31, 0.53] | -0.11 | 0.41 |
|  |  | Ukraine | 10 | 0.47 | 0.08 | [0.33, 0.62] | -0.04 | 0.98 |
|  |  | Uzbekistan | 5 | 0.47 | 0.05 | [0.38,0.56] | -0.04 | 0.85 |
|  | *p < 0.05 | | | | | | | |

Continued

| **Item** | | **Country** | **n** | $\hat{\boldsymbol{p}}$ | **SE** | **95%-CI** | **Effect** | **p-value** |
| --- | --- | --- | --- | --- | --- | --- | --- | --- |
| The collaboration with the telemedical specialist was always friendly and constructive | | Germany | 8 | 0.65 | 0.04 | [0.56, 0.72] | 0.19 | **0.04*** |
|  |  | South Africa | 7 | 0.36 | 0.08 | [0.22, 0.53] | -0.19 | 0.38 |
|  |  | Ukraine | 10 | 0.45 | 0.07 | [0.31, 0.59] | -0.07 | 0.85 |
|  |  | Uzbekistan | 5 | 0.55 | 0.08 | [0.39, 0.70] | 0.06 | 0.92 |
| During the rounds, the telemedical specialist treated my patients respectfully | | Germany | 8 | 0.52 | 0.08 | [0.37, 0.67] | 0.03 | 0.99 |
|  |  | South Africa | 7 | 0.46 | 0.08 | [0.31, 0.62] | -0.06 | 0.94 |
|  |  | Ukraine | 10 | 0.45 | 0.08 | [0.31, 0.60] | -0.06 | 0.91 |
|  |  | Uzbekistan | 5 | 0.57 | 0.08 | [0.41, 0.71] | 0.09 | 0.83 |
| My patients accepted the telemedical specialist very well | | Germany | 8 | 0.52 | 0.09 | [0.34, 0.67] | 0.01 | 0.999 |
|  |  | South Africa | 7 | 0.34 | 0.07 | [0.22, 0.49] | -0.21 | 0.21 |
|  |  | Ukraine | 10 | 0.47 | 0.08 | [0.33, 0.61] | -0.04 | 0.97 |
|  |  | Uzbekistan | 5 | 0.68 | 0.08 | [0.52, 0.81] | 0.24 | 0.19 |
| The telemedical rounds helped me in treating my patients | | Germany | 8 | 0.57 | 0.08 | [0.42, 0.71] | 0.10 | 0.75 |
|  |  | South Africa | 7 | 0.30 | 0.07 | [0.19, 0.45] | -0.26 | 0.12 |
|  |  | Ukraine | 10 | 0.46 | 0.07 | [0.33, 0.60] | -0.05 | 0.95 |
|  |  | Uzbekistan | 5 | 0.66 | 0.08 | [0.49, 0.79] | 0.21 | 0.29 |
| I have ethical concerns about telemedical visits | | Germany | 8 | 0.32 | 0.08 | [0.20, 0.49] | -0.23 | 0.23 |
|  |  | South Africa | 7 | 0.47 | 0.08 | [0.32, 0.63] | -0.04 | 0.97 |
|  |  | Ukraine | 10 | 0.57 | 0.08 | [0.42, 0.72] | 0.10 | 0.74 |
|  |  | Uzbekistan | 5 | 0.63 | 0.12 | [0.38, 0.83] | 0.18 | 0.68 |
| I have data protection concerns about telemedical visits | | Germany | 8 | 0.28 | 0.07 | [0.17, 0.42] | -0.30 | 0.10 |
|  |  | South Africa | 7 | 0.62 | 0.08 | [0.46, 0.76] | 0.15 | 0.47 |
|  |  | Ukraine | 10 | 0.52 | 0.08 | [0.36, 0.66] | 0.02 | 0.996 |
|  |  | Uzbekistan | 5 | 0.59 | 0.12 | [0.34, 0.80] | 0.12 | 0.86 |
| Overall, I am satisfied with telemedical rounds | | Germany | 8 | 0.52 | 0.08 | [0.36, 0.67] | 0.03 | 0.99 |
|  |  | South Africa | 7 | 0.42 | 0.08 | [0.27, 0.58] | -0.11 | 0.70 |
|  |  | Ukraine | 10 | 0.49 | 0.08 | [0.34, 0.65] | -0.01 | 0.999 |
|  |  | Uzbekistan | 5 | 0.57 | 0.10 | [0.38, 0.74] | 0.09 | 0.87 |
| All in all, I think there is still potential for quality improvement of the telemedical visits | | Germany | 8 | 0.39 | 0.10 | [0.22, 0.60] | -0.14 | 0.69 |
|  |  | South Africa | 7 | 0.41 | 0.07 | [0.28, 0.55] | -0.12 | 0.53 |
|  |  | Ukraine | 10 | 0.54 | 0.07 | [0.40, 0.68] | 0.06 | 0.90 |
|  |  | Uzbekistan | 5 | 0.66 | 0.11 | [0.41, 0.84] | 0.21 | 0.53 |
|  | *p < 0.05 | | | | | | | |
